# Breaching Medical Ethics in Research

**DOI:** 10.5812/traumamon.17112

**Published:** 2014-03-18

**Authors:** Mohammad Hosein Kalantar Motamedi

**Affiliations:** 1 Trauma Research Center, Baqiyatallah University of Medical Sciences, Tehran, IR Iran

**Keywords:** Ethics, Research, Medicine

As a researcher with several affiliations and commitments, I am often asked to assess clinical research studies, and approve student dissertation proposals prior to their initiation. One of the most common issues of concern which I often come across, is an unethical research methodology, that if conducted, would breach medical ethics in one way or another. This issue goes to show that the subject of medical ethics is often either misconstrued, downplayed, or ignored by students contemplating research in the medical field. We as physicians, are obligated to abide by and uphold ethical values and to relay them to our students. It is imperative that students contemplating clinical research, consider all aspects of medical ethics, both in their proposals and study design. They should be aware of what medical ethics is and what exactly constitutes a breach of medical ethics.

Medical ethics is defined as a system of moral principles that bring values and judgement to the practice of medicine. As a scholarly discipline, medical ethics encompasses practical application in clinical settings. Historically, Western medical ethics may be traced to guidelines relating to the duty of physicians that were formulated in antiquity, such as the Hippocratic Oath, and early religious teachings. The first code of medical ethics, ‘Formula Comitis Archiatrorum’, was published in the 5th century during the reign of the Ostrogothic king, Theodoric the Great. In the medieval and early modern period the issue of ethics is attributed to scholars of Muslim medicine, such as Ishaq ibn Ali al-Ruhawi (who wrote ‘Conduct of a Physician’, the first book dedicated to medical ethics), and Muhammad ibn Zakariya ar-Razi (known as Rhazes in the West), as well as other philosophers, such as Maimonides, Roman Catholic scholastic thinkers like Thomas Aquinas and case-oriented analysts (casuistry) of Catholic moral theology. These intellectual traditions were propagated and incorporated in Catholic, Islamic and Jewish medical ethics. By the 18th and 19th centuries, medical ethics had emerged as a more self-conscious discourse. In England, Thomas Percival, a physician and author, established the first modern code of medical ethics. He drafted a pamphlet with the medical code in 1794 and wrote an expanded version in 1803; he also coined the terms, ‘medical ethics’ and ‘medical jurisprudence’ (1).

The human rights era started with the formation of the United Nations (UN) in 1945. The UN pursued the global promotion of human rights and the Universal Declaration of Human Rights (1948) was the first major document published to define human rights. 

Medical doctors have an ethical duty to protect the patients’ human rights and dignity in research as well as treatment. Most codes of medical ethics also require respect for the patient’s human rights in publishing. In Europe, the Council of Europe promotes the rule of law and observance of human rights. In 1997, the Council of Europe adopted the European Convention on Human Rights and Biomedicine in order to create a uniform code of medical ethics for its 47 member-states. The convention applies international human rights laws to medical ethics. In addition, it also provides special protection for the physical integrity of those who are unable to consent, which includes children (2). In December 2013, 29 member-states of the Council of Europe ratified the convention (3).

The United Nations Educational, Scientific and Cultural Organization (UNESCO) also promotes the protection of dignity and human rights. According to UNESCO, ‘Declarations are another means of defining norms, which are not subject to ratification’. Similar to recommendations, they set forth universal principles to which the community of States wished to attribute the greatest possible authority and to afford the broadest possible support. UNESCO adopted the Universal Declaration on Human Rights and Biomedicine to advance the application of international human rights laws in medical ethics. This Declaration provides special protection of human rights for incompetent persons.

The Declaration of Helsinki is a set of ethical principles regarding human experimentation developed for the medical community by the World Medical Association (WMA). It is widely regarded as the cornerstone document of human research ethics (4, 5). Abiding by the ethical principles stated in the Declaration of Helsinki is now mandatory in most medical journals. Although, it is noteworthy that medical ethics covers a wide scope of topics, including duplication and plagiarism; however, the point I wish to emphasize here is the ethical concerns in clinical trials. Students submitting their research proposals need to consider several ethical concerns prior to initiation of their clinical study. The 10 most common issues which are frequently neglected when dealing with study proposals, which I would consider to be a breach of medical ethics in one way or another, are listed below:

1. Any procedure or intervention conducted on a patient that is not performed in the accepted routine management of that disorder, or which is unnecessary and/or potentially harmful.
2. Administration of a drug or medication that is not given in the normal management of a disorder, or which is unnecessary, potentially harmful or its effects are unknown.
3. Obtaining a signed consent in an attempt to justify an unethical procedure or immoral methodology.
4. Exposure of the patient to an invasive clinical procedure or a potentially harmful noninvasive procedure, in the interests of a study.
5. Exposure of the patient to undue radiation, no matter how small the dose, for the purposes of study, analysis, comparison, etc.
6. Attempting a new invasive human clinical procedure without prior successful animal studies and without institutional review board approval.
7. Testing new medications without prior successful animal studies, FDA approval, or well-documented indications for prescription.
8. Harvesting an organ or tissue from one who does not have the capacity to give consent.
9. Attempting paraclinical procedures deemed unnecessary by current documented studies.
10. Use of an inferior procedure when a better procedure is available and has been proven more effective, less invasive or with less complications, etc., for the sake of comparison.

To conclude, in the light of the aforementioned issues, students must check to ensure that the methodology of their study does not violate these basic ethical principles. They should also note that in applying and advancing scientific knowledge, medical practices and associated technologies, human weakness must be taken into account. Individuals and groups of special vulnerability should be protected and the personal integrity of such individuals must be respected (5). All medical and healthcare studies should conform to the global standards of medical ethics and must respect human rights.
